# Preferential transcription of the mutated allele in *NPM1* mutated acute myeloid leukaemia

**DOI:** 10.1038/s41598-020-73782-x

**Published:** 2020-10-19

**Authors:** G. D. Bailey, L. Doolan, A. Baskar, L. C. Smith, C. H. Seedhouse

**Affiliations:** Blood Cancer and Stem Cells, Division of Cancer and Stem Cells, School of Medicine, University of Nottingham Biodiscovery Institute, University Park, Nottingham, NG7 2RD UK

**Keywords:** Acute myeloid leukaemia, Reverse transcription polymerase chain reaction

## Abstract

Nucleophosmin is commonly both over-expressed and mutated in acute myeloid leukemia (AML). *NPM1* mutations are always heterozygous. In addition, *NPM1* has a number of different splice variants with the major variant encoded by exons 1–9 and 11–12 (*NPM1.1*). Further variants include *NPM1.2* which lacks exons 8 and 10 and *NPM1.3* which comprises exons 1–10 (and so lacks the region of sequence mutated in AML). In this study we quantified the expression of these three variants in 108 AML patient samples with and without *NPM1* mutations and also assessed the level of expression from the wild-type and mutant alleles in variants *NPM1.1* and *NPM1.2.* The results show that *NPM1.1* is the most commonly expressed variant, however transcripts from wild-type and mutated alleles do not occur at equal levels, with a significant bias toward the mutated allele. Considering the involvement of mutant nucleophosmin in the progression and maintenance of AML, a bias towards mutated transcripts could have a significant impact on disease maintenance.

## Introduction

Variation in gene expression between alleles occurs frequently across many genes in heterozygous individuals^1,2^. The resulting allelic imbalance (AI) has been shown to occur with particular frequency as a result of epigenetic changes which occur during development and as progenitor cells progress through differentiation^3,4^. The contribution of AI to disease progression from cancer driver mutations is not well understood. Acute myeloid leukaemia (AML) is a molecularly heterogeneous disease with diverse mutation backgrounds and pathways of disease progression^5^. Within this diverse genetic landscape, *NPM1* is the most commonly mutated gene in AML, occurring in around one-third of newly diagnosed patients^6^.

Nucleophosmin (NPM1), the protein encoded by *NPM1,* is a ubiquitous phosphoprotein which mediates numerous cellular functions (reviewed in^7^) and is considered essential for cell survival. NPM1 consists of a number of motifs, some of which mediate interactions with a range of binding partners, whilst others influence its cellular localisation. Although NPM1 contains signal motifs conferring cytoplasmic^8,9^, nucleoplasmic^10^ and nucleolar^11^ compartmentalisation, the protein is primarily localised to the nucleolus under normal physiological conditions.

Mutation of *NPM1* causes a frameshift in the 3′ region of the gene resulting in a protein with an abnormal C-terminus, leading to aberrant cytoplasmic localisation. Mutations of *NPM1* are always heterozygous and retain a wild-type allele^12^. To date, more than 50 different mutations in the *NPM1* gene have been identified^13^, all of which share certain characteristics. For example, with a small number of exceptions, mutations are restricted to exon 12 and result in an altered C-terminal region of the translated protein. As a consequence, mutated NPM1 loses nucleolar directing signals and gains a nuclear export signal^14^. Although these changes occur to different degrees between different mutations, the net result is a shift in the equilibrium of mutant NPM1 from the nucleolus to the cytoplasm^15^. Due to this, mutated NPM1 is designated cytoplasmic NPM1 (NPMc+) and the ability of the mutant protein to oligomerise with wild type protein results in both wild type and mutant variants located in the cytoplasm. Mutation of the *NPM1* gene is generally regarded to be a founder genetic alteration in AML and the presence of cytoplasmic NPM1 has been demonstrated to be required for disease maintenance^16^.

Mutation A is by far the most commonly represented, occurring in approximately 80% of cases of AML with mutated *NPM1*^17^. Mouse knockout studies have shown that a functional allele is required as embryonic lethality was observed in double *NPM1* knockouts^18,19^. Furthermore, a number of studies have demonstrated that mutation of a single allele is sufficient to induce myeloproliferation^20,21^. Several transcript variants of *NPM1* exist which arise through alternative mRNA splicing of the 12 exons that comprise the gene. None of the splice variants contain all 12 exons. The most characterised are *NPM1.1*, *NPM1.2*, and *NPM1.3* (NCBI Reference Sequence: NM_002520.6; NM_199185.3; NM_1037738.2 respectively). Of these, *NPM1.1* is the most frequently transcribed and well-studied splice variant of the gene. *NPM1.1* corresponds to the longest variant, encoding a 294-amino acid polypeptide from 11 exons, with exon 10 missing. Variant *NPM1.2* encodes a shorter polypeptide of 265 amino acids as a result of the loss of exon 8 and exon 10. The shortest variant, *NPM1.3*, lacks exons 11 and 12, but retains exon 10 and encodes a protein of 259 amino acids. This variant lacks 35 amino acids of the C-terminal region and is observed in the nucleoplasm^22^. For this reason, *NPM1.3* resembles mutated *NPM1*. Of these three variants, *NPM1.1* is considered to be the most biologically significant while the roles of *NPM1.2* and *NPM1.3* remain unclear^23^. Even though the *NPM1* mutation is always heterozygous in AML, little is known to what extent the two alleles are transcribed relative to each other.

In this study, we sought to identify whether the presence of mutated *NPM1* resulted in the differential transcription of the three splice variants. Using a panel of PCR primers we screened 108 patient derived AML samples. No difference was observed between variants *NPM1.2* and *NPM1.3*, however we found that transcripts derived from *NPM1.1* were expressed at approximately twice the copy number in samples with mutated *NPM1* than those with the wild-type gene. We identified that the elevated transcript copy number was due to an allelic imbalance between the mutated and wild-type allele.

## Results

### Validation of primer efficiency

**Figure 1:**
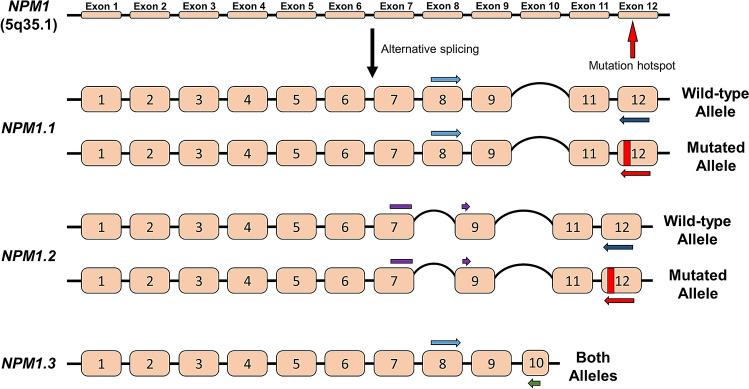
Schematic representation of the *NPM1* gene and the three main splice variants analysed in this study. Exons are denoted using numbered squares. The location of a TCTG duplication in exon 12 (mutation A) is indicated for variants *NPM1.1* and *NPM1.2*. Coloured arrows adjacent to coding sequences describe regions of primer binding allowing discrimination between each splice variant and wild-type and mutated alleles. The schematic was created using BioRender.

PCR primers were designed to discriminate between splice variants and between wild-type and mutated alleles (Fig. 1, Table1).

**Table 1:** Primer nucleotide sequences and combinations used to amplify *NPM1* splice variants with either wild-type or mutation A containing alleles.

Target	Sense strand 5′-3′	Antisense strand 5′-3′
*NPM1.1* wild-type	CAACACCAAGATCAAAAGG	CCTCCACTGCCAGAGA
*NPM1.1* mutated	CAACACCAAGATCAAAAGG	CTTCCTCCACTGCCAGACAGA*
*NPM1.2* wild-type	GAAGAAAGGACAAGAATCC	CCTCCACTGCCAGAGA
*NPM1.2* mutated	GAAGAAAGGACAAGAATCC	CTTCCTCCACTGCCAGACAGA*
*NPM1.3*	CAACACCAAGATCAAAAGG	CTGTTCAATGCGCTTTTTC

*Primer sequence taken from Gorello et al.^28^.

**Figure 2:**
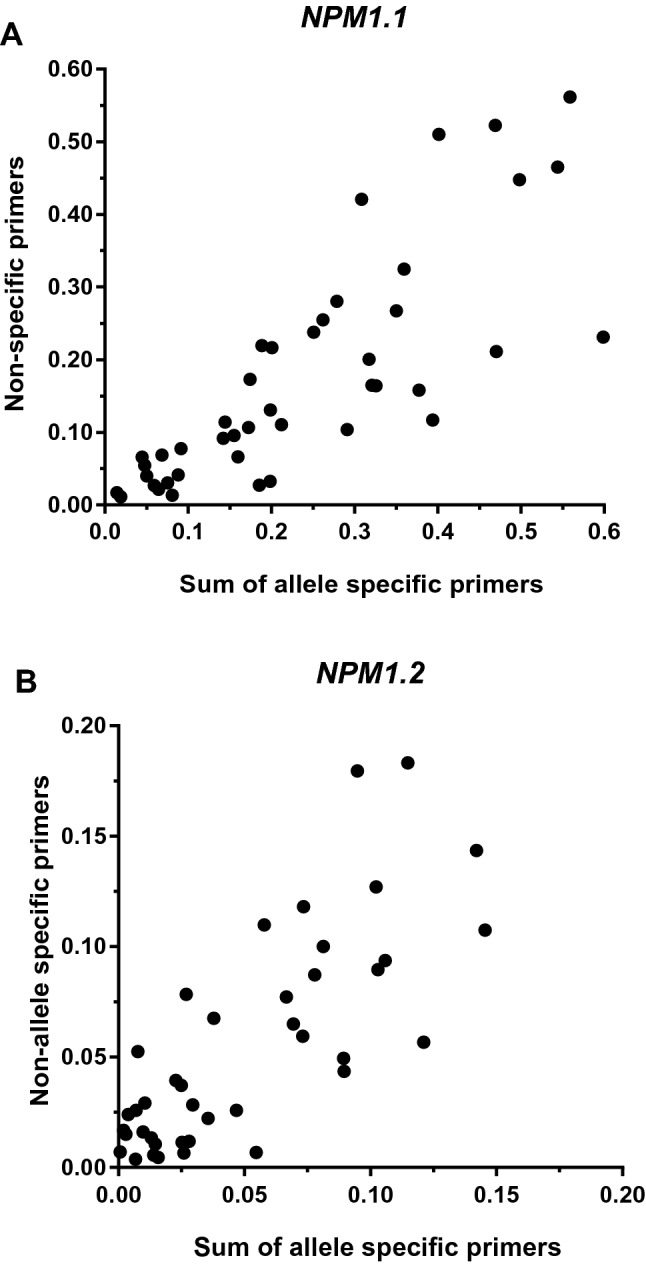
Spearman rank correlation of normalised transcript copy numbers quantified using non-discriminating and allele specific primer pairs. Samples from patient derived AML blasts containing an *NPM1* mutation were analysed (N = 44). (**A**) Correlation of transcript copy number for *NPM1.1*: R = 0.8482; *P* =  < 0.001. (**B**) Correlation of transcript copy number for *NPM1.2*: R = 0.7537; *P* =  < 0.001. Confidence intervals of 95% were assigned for each variant.

Transcript copy numbers for wild-type *NPM1* samples were measured directly whilst copy numbers for mutated samples were derived from the sum of wild-type and mutated copy numbers due to the heterozygosity of samples with the mutation. To validate that there was no difference in PCR efficiency between wild-type and mutated targets, *NPM1* mutated samples were used to obtain normalised copy number measurements with non-discriminating primer pairs and allele specific primer pairs. A Spearman rank correlation of 0.85 (*NPM1.1*) and 0.75 (*NPM1.2*) indicated equivalent PCR efficiencies for all primer combinations (Fig. 2).

### Quantitative analysis of *NPM1* splice variants

**Figure 3:**
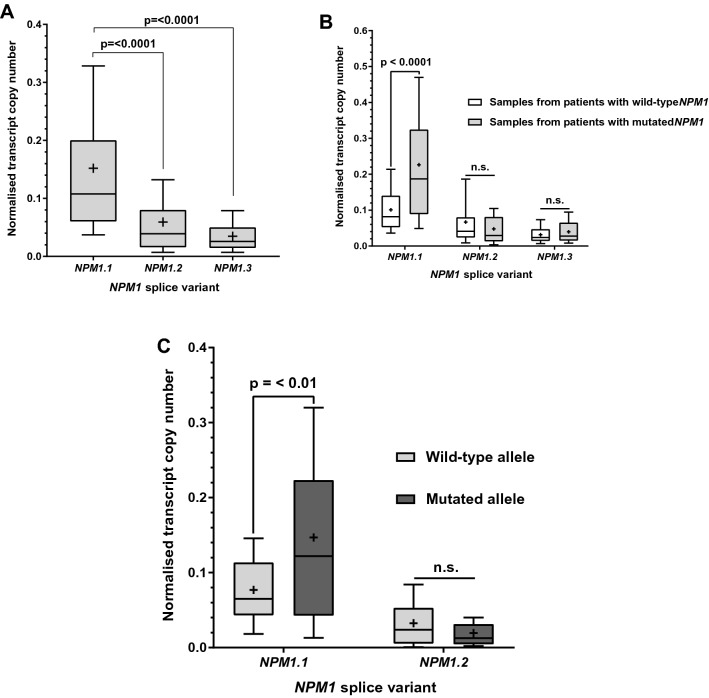
Analysis of NPM1 transcript copy numbers for the three main splice variants in a cohort of patient derived AML blasts, quantified using qRT-PCR. (**A**) Normalised transcript copy numbers were obtained for each of the splice variants in the total cohort (N = 108). (**B**) The samples were then separated into those containing either a wild-type *NPM1* allele (N = 64) or mutated allele (mutation A) (N = 44) and normalised transcript copy number calculated for each splice variant. (**C**) Samples with a mutated *NPM1* allele (N = 44) were next analysed for *NPM1.1* and *NPM1.2* variant transcripts derived from either the wild-type or mutated allele. For each data set, a Shapiro–Wilk test of Normality indicated the data were not normally distributed, non-parametric Mann–Whitney tests were used for statistical analysis. Whiskers show 10–90 percentile. Boxes denote 25–75 percentile. Median values indicated with a bar. Mean values indicated with a plus symbol.

The copy number of RNA transcripts encoding NPM1 in a cohort of 108 patient samples was measured using qPCR. A panel of novel PCR primers allowed the transcript copy number to be measured for each of the three most commonly occurring *NPM1* splice variants; *NPM1.1*, *NPM1.2*, and *NPM1.3*. The primers also allowed for the discrimination between transcripts encoded by either the wild-type or mutated allele (mutation A). Transcript copy numbers were calculated using standard curves prepared from serial dilutions of plasmids sub-cloned with each target sequence. All transcript copy numbers were normalised to a house keeping gene (beta 2-microglobulin). The normalised quantity of transcripts encoding each splice variant was calculated (*NPM1.1* median = 0.108, *NPM1.2* median = 0.039, *NPM1.3* median = 0.023 Fig. 3a). Transcripts for *NPM1.1* were the most abundant, present at 2.8X and 4.8X those encoding *NPM1.2* and *NPM1.3* respectively (*NPM1.1* vs *NPM1.2*; *P* =  < 0.0001; *NPM1.1* vs *NPM1.2*; *P* =  < 0.0001).

The sample cohort was then divided into *NPM1* wild-type and *NPM1* mutated (mutation A) samples and normalised transcript copy numbers for each splice variant calculated. Initially, transcript copy number for each splice variant in samples from patients with wild-type *NPM1* (N = 64) and those with mutated *NPM1* (N = 44) were compared (Fig. 3b)*.* The results demonstrated no difference in normalised transcript copy number between wild-type and mutated samples for splice variants *NPM1.2* and *NPM1.3* (*NPM1.2* wild-type median = 0.041, *NPM1.2* mutated median = 0.037; *P* = 0.4009: *NPM1.3* wild-type median = 0.025, NPM1.3 mutated median = 0.028; *P* = 0.2693).

Quantification of *NPM1.1* transcript copy numbers however, revealed that these transcripts were present at a 2.3 fold higher level in samples with a mutated allele than those with wild-type alleles (*NPM1.1* wild-type median = 0.082, *NPM1.1* mutated median = 0.19, *P* < 0.0001).

The patient samples with an *NPM1* mutation (N = 44) were then analysed for transcript copy numbers originating from either the wild-type or mutated allele (Fig. 3c) (splice variant *NPM1.3* was excluded from this analysis due to it lacking the mutated exon). Splice variant *NPM1.2* did not demonstrate a significant difference in normalised transcript copy number between the wild-type and mutated allele (*NPM1.2* wild-type median = 0.024, *NPM1.2* mutated median = 0.012; *P* = 0.0731). However, a 1.9 fold difference in transcript copy number in favour of the mutated allele for the *NPM1.1* variant was observed (*NPM1.1* wild-type median = 0.065, *NPM1.1* mutated median = 0.12; *P* = 0.0031). This clearly demonstrated an allelic imbalance biased towards the mutated allele which accounted for the excess of mutated transcripts relative to wild-type observed between samples with or without mutated *NPM1* demonstrated in Fig. 3b.

### Stability of NPM1 wild-type and mutated transcripts

In order to identify whether the observed difference in transcript copy number was due to an inherent increase in the stability of mutated transcripts, the half-life of wild-type and mutated transcripts was determined. OCI-AML3 cells were incubated with actinomycin D to inhibit transcription. Transcripts from the wild-type or mutated allele were measured using qPCR over a time course of 14 h (see Supplementary Fig. S1 online). The half-life of each target was calculated using linear regression of normalized transcript quantities. Half-lives of *NPM1* wild-type and mutated transcripts were determined as 9.14 and 9.54 h respectively (*P* = 0.895, N = 3) demonstrating no difference in transcript stability.

## Discussion

Mutation of *NPM1* is widely accepted to be a driver mutation in AML as well as being required for disease maintenance. Understanding the contribution that mutated *NPM1* makes to AML is therefore crucial to meet the challenges of developing new treatment strategies. The biological roles of *NPM1.2* and *NPM1.3* are not well understood and their contribution to the onset and maintenance of AML remains uncertain. A small number of studies however have revealed some insights into the role of *NPM1* splice variants in AML. Zajac et al.^24^, demonstrated that relative transcript levels for the three variants were higher in AML cells than in healthy control samples. Furthermore, in cytogenetically normal patients, a higher level of *NPM1.3* expression was associated with significantly longer overall survival compared to patients with low levels of *NPM1.3* (referred to as R2 in the study).

In a different study, Handschuh et al.^23^ showed that *NPM1.1* was the most highly expressed variant, at just over 30 times the level of *NPM1.2* and three times that of *NPM1.3*. In addition, Handschuh et al., demonstrated a significant decrease in the levels of *NPM1.2* in mutated samples compared to wild-type samples. In the patient cohort investigated during the present study, *NPM1.1* was the most highly expressed variant, however the magnitude of difference between the three variants was less than that observed by Handschuh et al. This could be explained by intrinsic differences between patient cohorts and the different methodologies employed by the two studies. From the small body of work that have addressed the roles of *NPM1* splice variants in AML, *NPM1.2* and *NPM1.3* are likely to be much less influential on disease progression and maintenance than *NPM1.1*.

Little is currently known about the role of AI in the development and progression of leukaemia with a paucity of literature on the subject. A recent study by Batcha et al.^25^, has addressed this issue. They demonstrated an AI in the expression of a number of recurrently mutated genes in AML using RNA-sequencing. Among the genes investigated was *NPM1*, but the results for this gene were conflicting. In their study, Batcha et al., examined two patient cohorts; a primary cohort and a validation cohort. Analysis of the primary patient cohort demonstrated an AI in favour of the mutated allele, but a small shift in AI to the wild-type allele was seen in the validation cohort. This inconsistency was attributed to technical limitations of the method used in combination with the small effect size seen in allele-specific expression of the gene. Using methods distinct to those employed by Batcha et al., we have also observed an AI in *NPM1* transcription biased to the mutated allele*.* Using qPCR with allele specific and splice variant specific primers on a UK patient cohort our work demonstrates AI with preferential transcription of the mutated allele, supporting the results seen in the primary cohort from the work of Batcha et al. The mutational status of the cell appeared to have no effect on the level of transcription from the wild-type allele, (data not shown) presumably due to the requirement of the cell for a population of normally functioning NPM1. The reason for the imbalance is not clear, however our results demonstrate that no difference in the rate of decay exists between *NPM1* wild-type and mutated transcripts. We are able therefore to conclude that the AI does not result from increased stability of mutated transcripts relative to the wild-type. Due to the relationship between the leukaemic phenotype and mutant NPM1, cells with increased NPM1 expression would possess a survival advantage. An AI biased to the mutated *NPM1* allele could therefore have significant implications on the maintenance of AML. NPM1 is located primarily in the nucleolus, due to a carboxy-terminal nucleolar localisation signal, but continuously shuttles between the cytoplasm and the nucleolus in order to carry out numerous cellular functions. Mutations in *NPM1* result in structural disruption of the carboxy-terminus, causing aberrant cytoplasmic protein localization which ultimately drives the leukaemic phenotype^15^. Recent work has demonstrated that the cytoplasmic mis-localisation of NPM1 results in the upregulation of several *HOX* genes which are implicated in the maintenance of a proliferative, leukaemic phenotype^16^. Methods of inducing the nucleolar relocalisation of NPM1, for example by inhibiting nuclear export^16^ or via DNA damage responses^26^ are therefore likely to have therapeutic potential. The mutated allele bias that we and Batcha et al., have demonstrated is likely to exacerbate the leukaemogenic effects of NPM1 in AML with mutated *NPM1*and could present a significant influence to the leukaemic burden.

## Methods

### Patient samples

Blood or bone marrow samples were obtained from AML patients presenting to Nottingham University Hospital. The East Midlands—Nottingham 1 Research Ethics Committee approved the study protocol (reference 06/Q2403/16). Written informed consent was obtained from the participants for sample collection and analysis in accordance to the Declaration of Helsinki guidelines.

Mononuclear cells were isolated from AML patient samples using a standard density gradient/centrifugation method and were stored immediately in RLT buffer at – 20 °C. *NPM1* mutations were identified as described in^27^. Only *NPM1* mutated samples containing mutation A were analysed in this study.

### Primer design

Primers were designed to discriminate between the three main *NPM1* splice variants, *NPM1.1, NPM1.2, NPM1.3*. Sequence data for the three isoforms were obtained from the National Centre for Biotechnology Information and aligned using Molecular Evolutionary Genetics Analysis 7.0 software. Primers were designed in such a way as to also discriminate between wild-type and mutated alleles. The mutation A specific reverse primer sequence was taken from^28^. Iterative rounds of primer optimisations to test specificity and sensitivity were undertaken using conventional PCR before identifying the final sequences (Table 1). The generation of a single amplicon was confirmed by the production of a single peak during qRT-PCR dissociation curve analysis and by DNA sequencing.

### RNA extraction and reverse transcription

Extraction of RNA was achieved using a QIAamp RNA Blood Mini kit (Qiagen, Hilden, Germany) according to the manufacturer’s instructions. Reverse transcription was carried out with SuperScriptIII First-Strand Synthesis System using random hexamer primers and following the manufacturer’s recommended procedures. Following quantification, 2 µg of RNA was reverse transcribed into 30 µL of cDNA and diluted at a ratio of 1:20 with water for future applications.

### Generation of plasmid standards

Plasmid standards were constructed to allow for transcript copy numbers to be quantified using qRT-PCR. Target sequences for *NPM1.1* (wild-type and mutated), *NPM1.2* (wild-type and mutated) and *NPM1.3* were PCR amplified from OCI-AML3 cells using the primers described in Table 1. Amplified products were screened using gel electrophoresis and sequenced to confirm the presence of the correct product before sub-cloning into the pGEM-Teasy vector. Following sub-cloning, target sequences were validated using M13 forward and reverse primers in separate reactions. Plasmids were transformed into JM109 competent *E. Coli* cells, and stored in 40% glycerol at − 80 °C. Plasmids were isolated from *E Coli* cultures grown from resuscitated glycerol stocks using a plasmid mini isolation kit (NEB, Massachusetts, USA) and 2 ug of plasmid DNA was linearised using 10,000 units/mL of SphI. Plasmid copy numbers were calculated using the following equations:

1. To calculate the mass of plasmid plus insert:
  $$m=\left[n\right] \left[\frac{1mole}{6.023e23}molecules \left(bp\right)\right]\left[\frac{600g}{mole}\right]=\left[n\right]\left[1.096e-\frac{21g}{bp}\right]$$
  where m = mass, n = plasmid size (bp), 6.023e23 molecules per mole = Avagadros number and 600 g/mole = the average molecular weight of a double stranded DNA molecule.
2. To calculate plasmid copy number:
  $$Copies \,per \,\mu L=\frac{\left(6.023e23 \,copies\right) \times (plasmid \,concentration \,g/\mu L)}{(number \,of \,bases \times (660 \,daltons/base)}$$

### Quantitative RT-PCR

Transcript copy numbers were measured using a 7500 Fast Real-Time PCR analyser. Reaction mixtures were assembled using Fast SYBR Green Master Mix according to the manufacturer’s protocol. Samples were tested in triplicate and beta 2-microglobulin was used as a reference gene for data normalisation. Standard curves were constructed using tenfold serial dilutions of plasmids from 3 × 10^6^ to 300 plasmid copies per well. Quantity mean values were calculated using 7500 software v2.7 and presented using GraphPad Prism v7. All raw data are accessible via Figshare; 10.6084/m9.figshare.13066961.

### Statistical analysis

All statistical analysis and data plots were carried out using GraphPad Prism v7. Shapiro–Wilk tests were used to indicate distribution of the data. Non-parametric Mann–Whitney tests were used for statistical analysis. Confidence intervals were set at 95% of the mean. Non parametric Spearman rank correlations with 95% confidence intervals were calculated to test the correlation between mutant transcript copy numbers derived from non-discriminatory primers and the sum of allele specific primers.

## Supplementary information

Supplementary information

Supplementary figure

Supplementary figure
