# Supplementary material for: Preferential transcription of the mutated allele in NPM1 mutated acute myeloid leukaemia
Source: Sci Rep. 2020 Oct 19;10:17695. doi: 10.1038/s41598-020-73782-x (PMC7572395; doi:10.1038/s41598-020-73782-x)
Supplement: Supplementary file 1 — Supplementary information [file 41598_2020_73782_MOESM1_ESM.docx]

**Supplementary Method**

**mRNA decay analysis**

Transcript stability was assessed using the OCI-AML3 cell line. Cells were cultured in RPMI-1640 supplemented with 10% FCS and 1% L-glutamine. Cultures were maintained at 37°C in 5% CO_2_. Cells were treated with actinomycin D to prevent transcription. Actinomycin D was reconstituted in RPMI-1640 at a concentration of 0.5mg/mL. OCI-AML3 cells were seeded at 5X10^5^ cells per mL and treated with 10µg/ml Actinomycin D and incubated for 0, 4, 6, 8, 10, 12, and 14 hours at 37°C in 5% CO_2_. RNA was extracted using a QIAmp RNA Blood Mini kit (Qiagen, Hilden, Germany) according to the manufacturer’s instructions. Reverse transcription, using random hexamer primers, was carried out with SuperScriptIII First-Strand Synthesis System following the manufacturer’s recommended procedures. 2µg of RNA was reverse transcribed into 30µL of cDNA and diluted at a ratio of 1:20 with water for in preparation for analysis using qPCR.
