# Supplementary material for: Preferential transcription of the mutated allele in NPM1 mutated acute myeloid leukaemia
Source: Sci Rep. 2020 Oct 19;10:17695. doi: 10.1038/s41598-020-73782-x (PMC7572395; doi:10.1038/s41598-020-73782-x)
Supplement: Supplementary file 2 — Supplementary figure [file 41598_2020_73782_MOESM2_ESM.docx]

**Supplementary Figure S1**

Relative expression of *NPM1* following actinomycin D treatment. OCI-AML3 cells were incubated with actinomycin D (10μg/mL). RNA was extracted after 4, 6, 8, 10, 12 and 14 hours of incubation with the drug and transcript quantities measured using qPCR and normalised to a reference gene. Mean transcript quantities are expressed as a proportion of transcript quantity at 4 hours post treatment. Data points for each decay curve are shown (wild-type: square symbols; mutated: triangle symbols). Best fit lines for linear regression are shown for each target (wild-type: dashed line; mutated: dotted line). Error bars represent the standard error of the mean from three independent experiments (P = 0.895).
